# How general practitioners perceive the aging trajectory of oldest-old - A qualitative study

**DOI:** 10.1186/s12875-023-01964-3

**Published:** 2023-01-09

**Authors:** Emile Escourrou, Thomas Joyeux, Matthieu Guilhem, Stéphane Oustric, Virginie Gardette

**Affiliations:** 1 Département Universitaire de Médecine Générale, Faculté de Médecine Rangueil, Université Paul Sabatier Toulouse III, 133 route de Narbonne, 31400 Toulouse, France; 2 Maintain Aging Research team, CERPOP, Université de Toulouse, Université Paul Sabatier, Inserm, Toulouse, France; 3 Maison de Santé Pluriprofessionnelle Universitaire La Providence, Toulouse, France; 4 Centre Hospitalier Universitaire de Toulouse, Toulouse, France

**Keywords:** Age 80 and over, Aging, General practice, Independent living, Longevity, Qualitative research

## Abstract

**Background:**

A new population of older people is growing: the oldest-old. The care of the oldest-old (individuals aged 90 and over) is a new challenge in primary care. This study aimed to analyze the perception of General Practitioners (GP) on (1) the aging process of their patients up to a very advanced age, (2) how to adapt their practice to the care of these patients.

**Methods:**

We conducted a qualitative study using focus group (face to face) and individual (video call) interviews of GPs in southwest France. The sampling was purposive. We analyzed the interviews using an inductive approach based on the phases of thematic analysis. We used researchers’ triangulation during the process. Collection was concluded when saturation was reached.

**Results:**

Three focus groups and one individual interview were conducted with a total of seventeen general practitioners. GP perception concerning aging and very advanced age were based on their personal experience and their daily clinical practice. Aging was perceived as an individual, unconscious, unpredictable and irreversible phenomenon. The shift towards “very old age” appeared inevitable. It could be a physical or psychological shift, or patients neglecting themselves or lacking a project. The care of the oldest-old became more specific and individual, adapted to the wishes of the patient. Those adaptations involve medical disengagement to focus on the most essential outcomes. The objectives of health care needed to be less strict with limited invasive practices. Prevention needed to focus mainly on prevention of falls and limitation of functional decline.

**Conclusion:**

GPs identified an inevitable and unpredictable shift from old age to very old age. The adaption of the theory of disengagement allowed us to identify a medical disengagement of the GPs in the care of their oldest patients.

## Introduction

Between 2020 and 2040, the number of people aged 80 and over is expected to double [1]. Until 2050, this population is expected to grow from 126.5 million to 446.6 million worldwide [2]. Every continent will face this demographic evolution, more particularly the high- and medium-income countries [2].

This demographic evolution result from changes in lifestyle and in the leading causes of disease and death [3]. With the progress in infectious medicine and changes in lifestyle, noncommunicable diseases have replaced infectious diseases [3, 4]. Noncommunicable diseases affecting older people are the biggest burden on global health. Changes in lifestyle and diet are directly correlated with the increase of these chronic noncommunicable diseases (diabetes, heart diseases, cancers for example) [3, 4].

General population aging creates challenges in the health care of older people, from a medical, environmental and societal point of view. Adopted by medical and political organizations [5], the concept of successful aging has been proposed for the first time in 1987 by Rowe and Khan [6] and has become an objective in the care of older patients. The World Health Organization (WHO) defined it as a process of development and maintenance of functional abilities which allows older persons to enjoy a state of well-being [4]. It is a multi-dimensional approach, based on functional, psychological, economic and environmental care of the individual [4]. This objective necessitates a reorganization and adaptation of health care adapted to this population which increasingly expresses a wish to be cared for at home [7].

General Practice was defined by WONCA in 2002 like a clinical specialty oriented towards primary care and using a holistic person-centered approach [8]. Alongside geriatric medicine, it is the most adapted medical specialty to treat very old patients according to the bio-psycho-social model [9].

Various studies have explored this relationship between GPs and their older patients. On one hand, older patients expect from their GP a global, empathic approach, sensitive to the difference between task performance and affective performance [10, 11]. On the other hand, GPs strongly favor a collaborative approach based on information sharing with geriatric institutions [12]. Difficulties in the management of these patients are multiple, ranging from difficulty to evaluate the social status of the patient [13] to a collaborative approach to decision-making combining medical objectives and patient preferences [14].

Even though there is evidence on what patients and GPs expect, studies assessing GP perceptions and experiences related to the care of the oldest-old are lacking. As we consider these elements relevant for the health care of this category of patients, we addressed them in the present study.

The present study was included in global research focusing on the aging trajectory of the oldest-old. Our research question was: what are the key moments in the aging trajectory from old age to very old age? The aging trajectory (part of the life course) could be summarized as what people foresee for themselves and the process by which they construct and revise their subjective aging trajectory [15–17].

In a previous qualitative study, we first explored the perceptions of older people of aging through their life course. After analysis, we identified a shift from old age to very old age [18].

In the present study, we aimed to propose a different perspective of the life course of the oldest old through the perception of GPs. We hypothesized that this shift from old age to very old age was noticed by GPs during the follow up of their older patient.

The perceptions of both old patients and GPs at this crucial moment in the life course from old age to very old age could help to take it into consideration in care settings. It could lead to a better understanding of what oldest-old patients experience and to specific actions to prevent the negative effects of this shift.

The primary objective was to analyze the perception of GPs concerning the aging of their patients until very old age. The secondary objective was to document the experience of the GPs on how they adapted the care of patients to this life stage.

## Materials and methods

We chose to conduct a qualitative study using focus groups from 2019 to 2021. Focus groups were conducted by focusing on the discussion and interaction between participants. This method was deemed to be the most adapted to studying the experience and impressions of GPs when their patients reach very old age [19, 20].

We completed these focus groups with an individual interview of a GP whose profile was not included during the focus groups. We used the COREQ Checklist as a support to provide relevant criteria on how the study should be conducted [21].

### Study population

The study focused on GPs exercising in the south-west of France. Inclusion criteria were: having a doctorate in primary care medicine; exercising a regular professional activity; exercising in the south-west of France. Exclusion criteria were: having a practice different from the standard GP practice in France e.g. (activity oriented mainly on a part of the population like children, or based on alternative practices like homeopathy) [22].

### Sampling methodology

Sampling was based on a purposive sampling technique [23, 24]. The most varied range of GP profiles was sought. We made the hypothesis that, in the sampling strategy, it was relevant to include certain characteristics in these profiles. The characteristics of GPs susceptible to affect their responses concerning the research question were: age, sex, mode of practice (individual practice, in association with one or more other GPs, in a group clinic or a pluri-professional clinic), place of practice (rural, semi-rural or urban), number of years in practice, presence or absence of complementary geriatric training, whether they supervised medical students in their practice. We also considered the characteristics of their patients’ population (size, age distribution, cultural or socio-economic differences), auto evaluated by the GPs at the beginning of the interviews or during the recruitment phase (Table 1).

**Table 1:** Characteristics of the sampling^a^

Participants	Age (years) Mean (SD)	Sex (Male: M / Female: F) n (%)	Years of practice Mean (SD)	Mode of practice n (%)	Location of practice	Patients characteristics (% older patients, estimated by the participant)	Complementary geriatric training (-: no / +: yes)	Trainer (-: no; I: yes in initial training, C: yes in continuing professional training)	Internship supervisor (- : no / + : yes)
All	46.7 (13)	Female: 7 (41) Male: 10 (59%)	17 (14)	Medical health center: 13 (32) Working as substitute: 1 (5) Geriatric clinic: 1 (5) Sole practitioner: 2 (11)	Urban: 8 (47) Semi rural: 7 (41) Rural: 1 (5)	NA	Complementary geriatric training : 3 (17)	No: 14 (82) Initial training: 1 (5) Continuing professional training: 3 (17)	No: 12 (71) Yes: 5 (29)
A1	61	M	32	Medical health center with 5 GPs and 2 nurses	Semi-rural	Older patients (20%)	-	-	+
A2	35	F	5	Medical health center with 5 GPs	Semi-rural	Older patients (30%)	-	-	-
A3	61	M	32	Medical health center of 3 GPs	Semi-rural	Older patients (40%)	-	-	+
A4	55	M	24	Medical health center of 3 GPs	Urban	Older patients (30%)	+	-	+
A5	40	M	5	Medical health center of 3 GPs	Urban	Younger patients (3%)	-	-	-
B1	29	F	1	GP working as substitute	NA	NA	-	-	-
B2	29	M	1	Geriatric Clinic	Urban	NA	-	-	-
B3	50	M	21	Medical health center of 4 GPs	Semi-rural	Younger patients (5%)	+	C	+
B4	70	M	40	Sole practitioner	Urban	Older patients (75%)	-	-	-
B5	68	F	38	Sole practitioner	Urban	Older patients (30%)	-	-	-
B6	44	F	15	Medical health center of 2 GPs	Urban	Younger patients (10%)	-	-	-
C1	48	F	17	Medical health center of 3 GPs	Urban	Older patients (30%)	-	-	-
C2	34	F	4	Medical health center : 3 GPs + speech-language pathologist + chiropodist	Semi-rural	Mixed patients	-	-	-
C3	50	F	NA	Medical health center of 5 GPs	Semi-rural	Mixed patients (20%)	-	-	-
C4	34	M	1	Medical health center of 4 GPs, project for pluriprofessional medical health center, Chairman of territorial community of health professionals	Rural	Younger patients (Missing data), patients from associated GPs in the same clinic	-	C	-
C5	55	M	34	Medical health center of 3 GPs, Nursing home coordinating doctor	Urban	Older patients (40%)	+	Professor of general practice : I, C	+
D1	31	M	3	Medical health center of 3 GPs	Semi-rural	Older patients (10%)	-	-	-

^a^The first focus group included participants A1 from A5, the second participants B1 from B6, the third one participants from C1 to C5. The individual interview was conducted with participant D1

The profiles sought were adapted using a preliminary analysis of data collected during each interview. This method allowed the inclusion of missing profiles or persons having not made their views sufficiently represented [24].

### Recruitment

Recruitment was initially from the personal network of researchers including colleagues, fellow members of training groups and health care networks. The recruitment was then extended through snowball sampling: we asked participants of previous focus groups if there was in their entourage a GP corresponding to the several profiles searched for.

Participants were contacted directly (by TJ, MG, or EE). An email was sent outlining the status of the researchers, the main theme of the research project, the modalities of the focus group, as well as proposed date and place. Recruitment for a single focus group stopped when sufficient GPs contacted responded positively to the invitation (5 to 6 participants).

### Design of the interview guide

The interview guide was designed using literature data and our research hypotheses [20]. The interview includes a first phase for participants to introduce themselves to each other and exchange around their age, number of years in practice and place of practice, professional development, current practice, and to auto-evaluate the characteristics of their patients’ population (size, age distribution, cultural or socio-economic differences).

The interview guide included the following four phases:

- Explore the GPs’ representations of the aging patient.
- Explore their experience and impressions of very old age.
- Propose a definition of very old age based on their experience and impressions previously expressed.
- Discuss adaptions in the care of this population.

The guide was revised after each focus group. We reformulated certain questions to aid the participants’ understanding and added questions to fill the research objectives.

An individual interview guide was adapted from the focus group guide with the same central theme explored.

### Data collection

Data collection was carried out during focus groups to allow GPs to share their experience and to establish group dynamics [19, 20].

The focus groups were conducted in a meeting room of a pluri-professional university health clinic (Maison de Santé Pluriprofessionelle Universitaire) in south-west France. An external moderator (BC) moderated the focus group in a semi-directive way [19, 20]. He was trained in conducting focus groups for previous studies. The researchers (EE, TJ, MG) introduced the study objectives and interview guide to him before the first focus group.

Researchers welcomed participants and introduced themselves as students (TJ and MG) and researcher (EE) conducting a research project on older patients in primary care. Participants were placed around a round table and provided with drinks and snacks. The focus groups were recorded with smartphones using a dictaphone app. The focus groups were concluded when the 4 themes predefined in the interview guide were covered.

Data collection was stopped when data saturation was reached [24].

### Analysis

The audio recording of each focus group and individual interview was completely transcribed into text, as faithfully as possible, while respecting anonymity. The facial expressions and gestures were also noted.

We used an inductive analysis method called thematic analysis [24, 25]. We did not use a qualitative analysis software. The notes taken during the focus groups and individual interviews and working meetings were recorded in an electronic notebook. The researchers referred to it throughout the analysis phase to enrich the conceptualization and the analysis. A triangulation of researchers (EE, TJ, MG) was conducted throughout the analysis phase.

The researchers’ first step was to read the full interviews to familiarize themselves with the audio and text content.

The second step was to translate the verbatim interviews into initial codes. Each idea encountered in the participants’ speech was translated into an initial code. A single sentence from a participant could contain several ideas, therefore be translated into several codes. The name of the initial code represented the main idea.

The initial codes were then grouped into categories. Each category grouped initial codes which participated in explaining and illustrating the general concept of the category. The concept was defined on the basis of the initial codes and the category’s title was given to each conceptual category created after an effort of conceptualization starting from the global idea.

Once all the categories were created, to-ing and fro-ing between empirical (scientific literature) and theoretical data (preliminary results of analysis) allowed to identify the main research themes which allowed us to respond to the research question. These themes were based on the conjunction of several categories.

Once the themes were identified, they were presented to external researchers to check whether the concepts were understandable. Afterwards, themes were fine-tuned in order to achieve consensus between researchers.

Finally, the links between each theme were analyzed and described. The description of the content of the themes and the links between them allowed us in fine to produce a response to the research question.

### Ethics

The participants were informed about the methods and objectives of the study. Following this, we collected their written consent on a consent form before the beginning of each interview.

We respected the anonymity of participants and persons cited while we transcribed each interview.

Participants were free to leave the study at any time without justification.

The favorable opinion of the *Comité Éthique du Collège National des Généralistes Enseignants* was received, recorded as number 190,919,122 obtained on the 15th of October 2019.

## Results

### Sampling

Three focus groups (focus group) and an individual interview were conducted. Out of forty-nine GPs solicited to participate in the study, seventeen eventually participated. Among the GPs declining, five cited too complex logistics, ten cited lack of time, three were not interested, and three did not respond, nine were not available at the proposed dates, two declined at the last moment. The participants’ mean age was 46.7 years (Table 1).

Focus groups took place from 17 to 2019 to 4 March 2020. They were completed by one individual interview with a GP who did not express their views during the focus groups. The individual interview took place as visio call due to the Coronavirus epidemic context. The result of this interview confirmed the previous analysis based on focus groups and did not give rise to any new perspective on the topic of interest.

The lengths of the focus groups were comparable, they lasted on average 83 min (92, 83 and 73 min for each group). The individual interview lasted 45 min.

We reached data saturation after the 3rd focus group. The individual interview confirmed data saturation.

Three major themes emerged from the qualitative data:

- “Perception of aging and very old age: between personal experience and daily clinical practice”.
- “The specificity of oldest-old patients imposed an adaptation of primary care”.
- “Medical disengagement and management of oldest-old patients”.

We propose an explanation of those themes in the sections below, based on the content of each subtheme.

### Perception of aging and very old age: between personal experience and daily clinical practice

#### The concept of aging

The perceptions of participants concerning their patients’ aging process was influenced by their own experiences and their daily clinical practice. This subjectivity in their judgment was conscious and accepted.

Aging was perceived as an individual, conscious, irreversible, unpredictable phenomenon, with an unavoidable ending. It was felt like a dark cloud hanging over each patient and affecting their life course.

*A2: « When they start to explain that they can’t carry certain tasks at home. They already begin to feel that they are aging. Before, they could do everything, they weren’t conscious [of aging]. They weren’t conscious that they had become old, and then… Small things, cleaning windows for example.»*.

Participants were unsettled by phases of acceleration and slowing down and the inherent variability in the aging process. Participants described their patients’ aging process as a clinical and physiological decline of their capacity. Advanced age was generally associated with a state of polypathology and a high level of dependency.

#### Individual age: convergence of chronological age and biological age

To characterize their aging patients, participants used chronological age as well as biological age. There seemed to be a duality between these two concepts. Participants disagreed at which chronological age patients could be considered elderly, and they preferred to use a range of ages. Chronological age was considered a necessary criterion as participants expected to see a physiological decline related to age. However, it was judged to not be sufficient considering the diversity of patients’ health status.

B6: *“I think we all have among our patients 70-year-old persons who are nearly at the end of life stage, while others are twenty years older and are, or seem to be, in great form; so, hmm, I don’t know if… age means anything after all.”*

The concept of biological age started to impose itself without being codified.

A2: *“It is more a physical state and pathologies and autonomy than… hmm… the age itself.”*

The main factors considered to evaluate biological age were those that they qualified as markers of aging. These factors could be physical: physical aspect (hair color, skin condition, etc.) and physical condition (movement, muscular performance, balance, vivacity); neuro-psychological: presence of cognitive impairment, neuro-sensory functioning; medical: current pathologies, clinical history, polymedication; or socio-environmental: level of autonomy and social life, retirement, widowhood.

The idea of individual age emerged, encompassing chronological age and weighted by biological age.

#### The Oldest-Old: a particular category of older patient

The existence of a category of very old patients, the Oldest-Old was not mentioned spontaneously by the participants, but was found in the analysis of their responses. The shift to very old age was described as an inevitable stage of aging. Even if GPs tried to anticipate this stage, it remained unpredictable. Most of the time, they could only acknowledge it. It was the starting point of the inexorable decline towards the last stage of life. This stage could present in different forms. It could be physical and lead to a loss of autonomy (a fall, an acute illness, decompensation from a chronic illness) or psychological (realization of one’s mortality, grief, loss of social usefulness). The apparition of negligence towards one’s appearance or health, the absence of a project or planning were also observed. Participants noted a progressive disengagement of their patients with society, until complete loss of perceived social usefulness.

B4: *“You see that, but… there’s still a future. Because like I said, you feel that you’re getting old when you don’t have a future, I mean… this is a long way away”.*

In the same way that they had difficulties characterizing their older patients, participants found it difficult to characterize their oldest-old patients. They defined oldest-old patients based on chronological age (around 90 years) and biological age, although their personal representation could include the character of the patients, their closeness to death, their opposition to health care and their disengagement. Patients were conscious of their advanced age and had to face the idea of their own death. They were attached to their own home and institutionalization was often associated with decline.

B3: *“Ah no, not necessarily, because I have very old people who are… who are super… super autonomous.”*

### The specificity of oldest-old patients imposed an adaptation of primary care

Redefining the posture of the GP…

Participants felt that they had to offer individual and specific care to their oldest-old patients. The care plan for the oldest-old was adapted to their living conditions (place of living, environment, surroundings, habits) and their health status (level of autonomy and pathologies).

During a consultation, the clinical exam needed to be even more thorough and attentive. GPs adapted complementary exams and increased their monitoring of these patients compared to younger patients in the same situation. They considered the chronological age of patients, and their life expectancy. These adaptations needed to respect the will of the patients and could trigger in them the consciousness of having reached very old age. B4: *“Well I see it because I care for a very old doctor who told me: ‘it’s curious, since I am 92 you treat me differently.’”*.

Participants chose to focus on maintaining quality of life and slowing down functional decline, following the principle of “*adding life to years rather than years to life*”. They were committed to accompany their oldest-old patients and prioritized quality of life (lifting restrictions) and comfort (treating pain and avoiding invasive measures). Prevention occupied a secondary position and was mainly based on preventing iatrogenicity and cardio-vascular accidents.

They saw it as essential to “deprescribe”. Adapting the prescription for these patients represented a complex intellectual exercise. Participants also focused their efforts on the prevention of falls, malnutrition, sarcopenia, vitamin deficiencies, loss of autonomy, cognitive decline. They insisted also on slowing down decline by maintaining their patients’ physical activity.

… in relation with the evolution of the patient’s attitude

Participants described some patients as serene towards their health care, to the point of being resigned. These patients requested to be accompanied. Others seemed pessimistic, preoccupied, and requested strict maintenance of the medical objectives in the hope to preserve good health. Participants reported being aware of discrepancies between their speech and their actions. They recognized that they were more serene in their practice when their patients were conscious of their very old age and adopted an expectative attitude.

A3: *“They know that they have passed a certain age threshold, and the time to die will come, so yes, it changes everything, completely…, everyone is more relaxed.”*

#### Constant management difficulties

Participants reported facing many difficulties. While it was overwhelmingly preferred, keeping a patient at home represented one of the biggest difficulties. This required an adaptation of the environment and a significant social burden. Lacking time, participants tried to keep their patients coming to their practice, and reserved home visits to exceptional cases. Difficulties related to cognitive pathologies could increase difficulties in maintaining patients at home. Even if detection of cognitive pathologies was effective, participants lacked enough time and resources to use such methods, and complained about the lack of therapeutic options.

The opposition of oldest-old patients to the care management proposed by GPs was associated with a lack of cooperation with the implementation of prevention measures and therapeutic education. There could be a rejection of institutionalization or of the implementation of human and material assistance in the home.

It was difficult for patients to accept changes in their mode of life. Even if admission in a retirement home could be of benefit to oldest-old patients, as it allowed more careful monitoring and availability of human resources, it was envisaged only as a last resort. Participants had a negative view of such establishments and associated them with unhappy aging. They reported that this step was difficult to accept for their patients, who refused it and associated it with proximity to death. They considered that their patients aged successfully if they could end their life in their own homes. A4: *“Hmm, there’s still a big difference between older patients ending their days in a retirement home and those who are lucky to stay at home. (…) For these patients, ending up un in an institution, it’s really… well. it’s like signing their death warrant*.”

Another difficulty expressed was in referring their oldest-old patients to other specialists and to hospital services. They explained this difficulty by “*a growing lack of hospital beds during the last thirty years*.” These difficulties could bring participants to get annoyed or elaborate strategies to find a hospital bed for their patients. This resulted in the feeling that the care of their oldest-old patients rested solely on primary care and GPs. They had to organize the care of these patients, and having to manage with heavy pathologies on their own. They felt that this role, which normally fell to specialists, was imposed on them by the circumstances. D1: “*You see they start to be a bit better cared for, we are the ‘multi-specialist’ of these patients. It’s true that it’s complicated.”*

#### The absence of a contrasting frame of reference

Participants felt ill equipped to manage their oldest-old patients. While they used various geriatric evaluation scores and concepts like “frailty” in their evaluation, they complained about the lack of recommended best practice adapted for oldest-old patients.

A1: « *and I was horrified some time ago to realize that there was nothing about recommendations, research about older patients: nothing!*” A1: “*All we do and all most people do is wing it! This has to be said.*” In response to this situation, participants said that they used empirical and uncertain evidence to care for their very old patients. They admitted that their management of these patients was mostly informed by their personal experience, their gut feeling and their representations, in a subjective way. Clinical experience allowed them to better face these difficulties. They based their approach on remaining life expectancy. They remained nevertheless conscious of the imperfection of this intuitive approach, and admitted that they could make errors of appreciation.

#### The doctor-patient relationship: towards a tripartite relationship

Despite all the reported management difficulties, a well-meaning relationship was maintained between GPs and their oldest-old patients. Participants felt affection and compassion for their patients. They described themselves as accompanying parties, who would bring empathy and pedagogy, while allowing their patients to be decision-makers. They recognized that oldest-old patients trusted their GPs to do what was best for them. Participants questioned themselves while caring for this category of patients. Faced with the advancing age of their patients, participants sought to include the patient’s main carer and to prepare them to the risk of the patient dying. As loss of autonomy progressed and cognitive troubles appeared, the oldest-old patients were not fully able to manage their own health care, and a tripartite relationship became established between the GP, the patient, and the carer. This was sometimes viewed as infantilizing.

### Medical disengagement and management of oldest-old patients

At the same time, participants described within their practice a succession of adaptations, that could be defined as medical disengagement. The objectives of health care needed to be less strict (for example regarding blood pressure level or HbA1c level), with limited invasive practices (less specialized consultations and complementary examinations). Prevention needed to focus mainly on prevention of falls and limitation of functional decline. Deprescribing was the best illustration: A5: “*Of course, but each drug often has a role… Well we see why it was prescribed, well, I find that it is intellectually difficult to say ‘well, this drug, let’s just put it aside*’’.

This GP-led medical disengagement was rational and thought out. They reasoned about the risk-benefit balance of each decision. Faithful to the doctrine *primum non nocere*, the objective of medical disengagement was to do no harm to the patients. Participants insisted on accompanying patients and avoiding iatrogenicity. Medical disengagement was justified by lesser stakes in the management of these patients as they had already exceeded their imagined life expectancy. They were however exposed to the opposition of their patients, as these same patients’ past experience rested on following the recommendations of their GP.

A2: “*Well it’s difficult to adapt because they are… we have drilled into them for years that they need to be careful, be careful, be careful… At the time when we lift the pressure because we think, well, there’s not much point, we’re going to be OK, they say: ‘but why, am old’? Ironical tone: ‘no, well, I mean… (…) in fact it’s just to remove some of the medication, so that you have less to take.’ Then, imitating the patient: ‘but why did we not do it earlier?’ Taking a lighter tone: ‘ah well, it’s difficult to explain’. Taking a more serious tone: ‘it’s difficult!’”*.

## Discussion

### Main findings and answers to research question

The participants’ perception of their patient’s aging was enriched by their practice. They reported basing their practice on objective and subjective medical markers of aging. Aging, an individual, unconscious, unpredictable and irreversible phenomenon, was perceived as a dark cloud hovering over the individuals, affecting their individual life course, with a great variability between individuals. Participants used two main concepts that could be complementary or opposed: chronological age and biological age.

The shift towards “very old age” appeared inevitably. It could be a physical or psychological shift, or patients neglecting themselves or lacking a project (Table 2).

**Table 2:** Factors and markers of the shift from old age to very old age

Physical	Physical decline leading to a loss of autonomy A fall, Acute illness, Decompensation from a chronic illness Apparition of negligence towards one’s appearance
Psychological	Realization of one’s mortality, Grief Expressing loss of social usefulness Absence of a project or planning in near future

It seemed that their care became more specific and individual, adapted to the wishes of the patient. The lack of guidelines and tools could force GPs to use empirical or instinctual approaches. This sometimes made them uncomfortable. The care of these patients was reported as becoming more and more time-consuming, complex and concentrated on primary rather than on secondary or tertiary care.

The patient-doctor relationship shifted progressively towards a tripartite relationship between patient-doctor-family, and palliative care was favored with the aim to maintain a satisfactory quality of life for as long as possible. Participants observed successive changes in their priorities which were triggered either by their patients and their carers’ disengagement, or by the GPs and their medical colleagues’ disengagement (a phenomenon we termed medical disengagement).

### Characterizing oldest-old patients

Participants based their perceptions of very old patients on their own experiences. It seemed like the age of the GP, a proxy for their experience, influenced their perception [26]. If they had to set a chronological age threshold to define Oldest-Old patients, participants situated this age around 90 years old. The 90 years threshold if consistent with the literature and particularly the age cut-off used to characterize Oldest-Old cohorts in recent studies [27, 28].

The distinction between chronological and biological age was difficult to make in clinical practice [29]. Biological age was a better predictor of phenotypical characteristics of the patient but necessitated a complex bio-medical evaluation [29]. Similarly, social sciences concern themselves with social age: the social organization of the life stages as well as the social uses of age [30]. A global evaluation of the older person seemed necessary to handle the two complementary concepts and adapt the GPs’ medical practice. Research conducted by the British Geriatric Society on a Comprehensive Geriatric Assessment could be translatable to the evaluation of an oldest-old patient (fragile or not) so as to understand their mental, physical and environmental specificities [31].

Finally, during the life course of older people, we uncovered in our study two concepts, the shift to irreversible decline, and the association between institutionalization and closeness to death, as also shown in the literature [32, 33].

### Deprescribing as a marker of medical disengagement

Participants found that deprescribing was a difficult but necessary exercise in the care of their oldest-old patients. The importance of deprescribing was justified by the prevalence of multimorbidity in their oldest-old patients who needed to take multiple medications [34]. This increased risks of iatrogenicity and undesirable side-effects due to drug interactions. Some factors influenced negatively a deprescribing approach such as the lack of tools and specific guidelines, the fear of consequences of deprescribing a medication [35, 36]. Conversely, some factors influenced positively deprescribing: the preferences of the patient, the lack of formal data on the indication of the medication [35, 36]. The type of medication was also a factor, with a greater facility in stopping preventive than pain relief medication [35].

### Implications for general practice: which concept to use in the absence of guidelines?

The absence of recommendations was another difficulty uncovered in the care of very old patients in our study.

Scientific data issued from randomized clinical trials that could feed Evidence Based Medicine imply only rarely older or very old patients. These studies generally focus on the progressive care of a pathology among hospitalized and younger patients.

These data can be difficult to extrapolated to a primary care context where the number of patients suffering from multiple pathologies is high [37]. Their care necessitates a global approach rather than one centered on an organ or a pathology. For the last 15 years since the emergence of the concept of fragility, new recommendations have been made for primary care, centered on a global approach for the older patient, and considering medical and environmental data [38]. Future research can bring further evidence on the appropriateness of measures proposed also for Oldest Old patients.

Despite the lack of specific guidelines for the care of older patients suffering from poly-pathologies [39], recommendations on a comprehensive and patient-centered approach are useful [40]. This approach starts from the priorities expressed by the person and allows them, via the acquisition of skills useful for their self-care, to express their preferences in terms of their primary care [41, 42]. The aim is to design a care plan co-constructed by the patient and the health care professional, or by the patient, their family, and professionals [41, 42]. This leads to an individual approach of health care respectful of the wishes expressed by the patient [41].

The conceptual bases of this approach were present in the interviews, but the concept itself and the means to achieve this goal were not clearly identified. The impact of a specific training proposed to GPs on the theme of comprehensive and patient-centered approach of the very old patient will need to be further evaluated. In the same line, the idea of better individualization of the care of the very old patient, the coupling of a geriatric evaluation with the global care of the very old patient suffering from polypathology, could be a way forward [43]. This could allow the GP to adapt closely their approach to the functional state of their patient as well as to their preferences. This could also lead to improved patient-doctor relationship [44] which itself influences health outcomes [44].

Considering the close environment of the patient also seemed essential. Connections to the local environment, people and place, along with good family relationships are key facilitators of successful health care of an oldest old patient [45].

### Strengths and limitations

#### Credibility

Credibility of the study is reinforced by time spent by the researchers in the field of study. A triangulation of researchers was in place throughout the study, with 3 researchers trained in qualitative methods: during their medical training (TJ and MG) and during a dedicated second year of Masters’ degree (EE). The opinion of outside researchers trained in qualitative research was also sought.

The main limitation was the absence of feedback to the participants who weren’t given the results of the study and could not comment on the faithful use of their ideas and perceptions.

#### Transferability

The sample of GPs was carried out with the aim to recruit a diversity of profiles in terms of the sociological phenomenon under study.

The study was however carried out in a single region of France. The specificities of the primary health care system in this region and its organization may have influenced the responses of participants. These specificities could be absent in the response of participants coming from different health care organizations.

#### Dependability

Our research process was logical and well recorded. The process was however not audited by external experts.

### Perspectives

Demographic changes lead to a global reflection on the components of the medical decisions for old and very old patient (oldest-old). Their medical decision in old age has to take into account that a significant proportion of their patients can live to a very old age. In very old age, GPs need scientific data to adapt their decisions in preventive and curative care.

The inclusion of oldest-old patients in clinical trials could help to obtain specific data for this population. At present, the inclusion criteria in trials are mostly based on age but factors of the shift to very old age could be used as some complementary criteria to include the oldest-old in a holistic definition.

The impact of multi-domain intervention at a later stage in the life course (very old age) should also be studied. The results of such studies would guide the interventions needed at these ages.

From our results, we also identified further research needed:

- To complement our understanding of the aging trajectory and the shift from old age to very old age, the perceptions of primary care givers and health professionals involved in home care should be explored. The comparison with GPs’ perception and older people’s perception from previous studies could lead to a global understanding of the key steps in the life course of older patients.
- The medical disengagement described in our study should be discussed in extensive and specific research.
- Our results revealed the perceptions of GPs of their care of oldest-old patients but to our knowledge there isn’t any data on actual medical practice in primary care for this population. A complementary approach with an epidemiologic study could be proposed.

## Conclusion

The perception of GPs on the aging of their patients was enriched by their practice, and rested on objective and subjective medical markers of aging, as well as on two inter-dependent concepts: chronological and biological age. Participants agreed on the appearance of a shift towards very old age. The care of these patients became more specific and individual, adapted to the wishes of the patients. Participants observed successive modifications in their practice, triggered by either the patient and their family, or the GP and their specialist colleagues.

The adaption of the concept of disengagement described previously allowed us to identify a new concept: medical disengagement. This is characterized by a prioritization of health care on the objectives defined as essential by the patient, their relatives, and healthcare professionals, while medical acts that had been considered essential until then are abandoned.

The concept of medical disengagement helps in understanding the mechanisms potentially used by GPs to provide an adapted care. The validity of this concept should be confirmed with dedicated studies, especially longitudinal studies. Then, the awareness of this concept could help the GP to share the components of their medical decision with the patient.

The lack of data from scientific practice specific to the very old age implies a lack of recommendations. The oldest-old have to be included in clinical trials and multi-domain intervention to give the community scientific data needed. Meanwhile, a comprehensive and patient centered approach seems all the more a necessary adaptation of the care plan to the wishes of the very old patient.

## Data Availability

The data analyzed during the current study are available from the corresponding author on reasonable request.
